# Supplementary material for: Chromothripsis during telomere crisis is independent of NHEJ, and consistent with a replicative origin
Source: Genome Res. 2019 May;29(5):737–49. doi: 10.1101/gr.240705.118 (PMC6499312; doi:10.1101/gr.240705.118)
Supplement: Supplemental Material [file supp_gr.240705.118_Supplemental_file_1.zip › contigs/annotated_contigs/DB56/contig.2.DB56_length_428_mean_cov_3.69158878505.docx]

**DB56_length_428_mean_cov_3.69158878505**

```
ATTTTAATTTTAATGAAGTCTAGTTTATCAATTCTTTCTTTCATGACCATGCCTTTGGTGTTGTATCTAGACCCAAGGTCATATAGACT
 >chr13:37793226-37793470 + E=2e-135
TCTCCTGTTATTTTCTAGAAGTTTTATAGTTTTGTAATTTACAGTTAGGTCTATAATTGATTTTGAGTTAATTTTTGAAGGATGTAATA

TCTGTGCCTTTATTTATTTATTTATTTTTTGCATGTGGCTATCCAGCTGTTTCAGCATCATTTGT|G|TAATGGTGGGATAATGTAATT
                                                                  >chr13:37878547-37878
TTTCTCTCATTAATATCTCTTTTGATCTGTAAATAATACATTCCTCCACATTCTATATTAAACCCAGAAATAAAATCACAATGTTAATT
732 + E=8e-100
AAAGAGAAAGTGAGGAGAATAATTTCTAACATGTTGAAGGCACAGAATAAACTTTAGAGAATTATTAATATGTC
```
